# Alcohol use and its association with suicide attempt, suicidal thoughts and non-suicidal self-harm in two successive, nationally representative English household samples

**DOI:** 10.1192/bjo.2022.594

**Published:** 2022-11-03

**Authors:** Sarah Ledden, Paul Moran, David Osborn, Alexandra Pitman

**Affiliations:** Division of Psychiatry, University College London, UK; Centre for Academic Mental Health, University of Bristol, UK; and National Institute for Health Research, Biomedical Research Centre, University Hospitals Bristol NHS Foundation Trust and University of Bristol, UK; Division of Psychiatry, University College London, UK; and Camden and Islington NHS Foundation Trust, St Pancras Hospital, UK

**Keywords:** Suicide, alcohol disorders, epidemiology, self-harm, depressive disorders

## Abstract

**Background:**

Alcohol use is a risk factor for suicidal behaviour, yet the nature of the relationship is unclear. Most research on the topic is conducted in clinical populations, with few studies exploring this association across the general population.

**Aims:**

We investigated the association between specific domains of alcohol use and suicide attempt, suicidal thoughts and non-suicidal self-harm in a general population sample.

**Method:**

A total of 14 949 adults who completed the 2007 or 2014 Adult Psychiatric Morbidity Survey were included. We measured alcohol use with the Alcohol Use Disorders Identification Test (AUDIT). Domains of alcohol use relating to risk categories, weekly consumption, binge drinking, dependence symptoms, harmful effects and concern from others were derived from relevant AUDIT items. Self-reported past year suicide attempt, suicidal thoughts and non-suicidal self-harm were measured with the Clinical Interview Schedule, Revised.

**Results:**

We found a linear association between total AUDIT score and outcomes. Three of six specific domains of alcohol use (dependence symptoms, harmful effects of drinking and binge drinking) were associated with increased odds of all three outcomes. There was no association of outcomes with the other domains of alcohol use.

**Conclusions:**

We found evidence of a linear association between total AUDIT score and suicide attempt, suicidal thoughts and non-suicidal self-harm in a representative English general population sample. Our analyses suggest that where alcohol use significantly disrupts day-to-day functioning, this may underpin the relationship between alcohol use and suicide-related outcomes to a greater extent than higher alcohol consumption. Longitudinal research is needed to further understand these relationships.

Alcohol use is an established risk factor for suicidal behaviour both at the individual and population level.^1–5^ Yet we know little about the relationship between alcohol use and suicidal and self-harming behaviour in the general population, beyond diagnostic levels of disordered or harmful alcohol use.

Alcohol use disorders are associated with increased risk of non-suicidal self-harm,^6^ suicidal ideation,^7^ suicide attempts^8^ and suicidal death.^9^ Recent studies of this association indicate that there are likely potential causal mechanisms between alcohol use disorders and suicidal behaviour, even after accounting for genetic and familial environmental confounding.^10,11^ It is not clear, however, if increasingly harmful alcohol use and alcohol-related behaviours across the general population show proportionate associations with suicidal behaviour. Only one study^12^ to date looked at an association between increasingly harmful alcohol use and suicidal behaviour in an adult general population sample. This Korean-based population survey reported evidence that increasingly harmful alcohol use, as measured by categorised Alcohol Use Disorders Identification Test (AUDIT) score, was associated with suicidal ideation and suicide attempt in women, but not men. Others have inferred associations between harmful alcohol use, as measured by increasing alcohol consumption, and suicide, with mixed conclusions.^13,14^

Many studies investigating alcohol use and suicidal behaviour in the general population use binary risk groups to describe alcohol use,^15,16^ but this line of enquiry does not clarify how levels of disordered or risky alcohol use and alcohol-related problems relate to suicidal and self-harming behaviour. Although some studies have adopted more nuanced measures of alcohol use and suicidal behaviour, they have relied on samples of older or middle-aged adults,^13,14,17^ or individuals presenting to hospital following suicidal behaviour,^7,15^ and are therefore prone to selection bias. Furthermore, many studies in the field fail to adjust for comorbid psychiatric conditions,^13,14,17,18^ which is an important potential confounder of the relationship between alcohol and suicidal behaviour.^19^

## Aims

In this study, we sought to shed light on the relationship between alcohol use and suicidal behaviour in the general population by adopting a broad definition of suicidal behaviour, encompassing suicide attempts, suicidal thoughts and non-suicidal self-harm. Our primary aim was to investigate whether there is a linear association between increasingly harmful alcohol use, as measured by the AUDIT, and suicide attempt, suicidal thoughts and non-suicidal self-harm in a general population sample. Our secondary aim was to investigate the association of specific domains of alcohol use with these outcomes, to better understand the potential mechanisms linking alcohol use and suicidal behaviour. Alcohol use disorder and suicidal behaviour are both associated with distinct gender^11,20^ and age differences,^21^ and we therefore sought to determine whether the association between alcohol use and suicidal behaviour also reflected these differences.

## Method

### Study sample

We analysed data from the 2007 and 2014 Adult Psychiatric Morbidity Survey (APMS). APMS is a cross-sectional English household survey intended to measure the prevalence of mental ill health and treatment use in the general population. Every 7 years, a new randomly selected sample is invited to participate. The APMS sample comprises adults aged 16 years and older living in private households in England, and uses a stratified random probability sampling design to recruit a nationally representative sample, as previously described.^22^ Data collection occurred from October 2006 to December 2007 (2007 survey), and May 2014 to September 2015 (2014 survey). The overall response rate for collection was 57%, with a final sample of 14 949 respondents. We conducted analyses on the full sample, imputing missing data by using multiple imputation by chained equations. Pseudo-anonymised data is available to approved researchers, and was retrieved from the UK Data Archive. The authors assert that all procedures contributing to this work comply with the ethical standards of the relevant national and institutional committees on human experimentation and with the Helsinki Declaration of 1975, as revised in 2008. Ethical approval for the 2007 APMS study was obtained from the Royal Free Hospital and Medical School Research Ethics Committee (ethical approval reference number 06/Q0501/71), and ethical approval for the 2014 APMS study was obtained from the West London National Research Ethics Committee (ethical approval reference number 14/LO/0411). Verbal consent was witnessed and formally recorded to take part in phase 1 of the survey, and written informed consent was obtained from all participants for phase 2 contact and data linkage. Further information about the APMS collection and methods are described elsewhere.^22^

### Exposure variables

We defined our main exposure measure (harmful alcohol use) and six additional measures (derived from the AUDIT tool). These captured risk categories (low-/moderate-/high-risk alcohol use) and other aspects of harmful alcohol use behaviour: drinking quantity and frequency, binge drinking, dependence symptoms, harmful effects of drinking and concern from others about drinking.

Harmful alcohol use was measured with scores on the ten-item AUDIT.^23^ The AUDIT assesses alcohol consumption, drinking behaviours and alcohol-related problems over the past year, and was designed as a screening tool to identify and describe a broader spectrum of problematic drinking than alcoholism or alcohol use disorder.^23^ A list of AUDIT items is provided in Supplementary Table 1 available at https://doi.org/10.1192/bjo.2022.594. For our primary analyses, we used total AUDIT score as a continuous measure.

Risk categories were captured by categorising AUDIT scores into clinically meaningful risk categories, as consistent with World Health Organization guidelines,^24^ such that a score of 0–7 was considered ‘low-risk alcohol use’, a score of 8–15 was considered ‘moderate-risk alcohol use’ and a score of ≥16 was considered ‘high-risk alcohol use’.

Drinking quantity and frequency was derived from two AUDIT items relating to the frequency and quantity of drinking, both using range-based categories. Using these two variables, we derived a product that used mid-range values to estimate the average number of drinks consumed in a typical drinking week, in an approach suggested by Berlin et al.^25^ Estimated average drinks consumed per week were then grouped such that zero to six drinks is categorised as ‘light drinking’, seven to 13 drinks is ‘moderate drinking’, 14–20 drinks is ‘hazardous drinking’, 21–30 drinks is ‘harmful drinking’ and >30 drinks is ‘probable dependence’.^26^

Binge drinking was measured by a single AUDIT item: ‘How often do you have six or more drinks on one occasion?’. Responses were grouped as an episode of binge drinking ‘less than monthly or never’, ‘monthly’, ‘weekly’ and ‘daily or almost daily’.

Dependence symptoms were measured by summing the scores of three AUDIT items that asked about inability to stop drinking, failure to meet normal expectations because of drinking and feeling a need for drink after a heavy session. Scores for dependence symptoms ranged from 0 to 12.

Harmful effects of drinking were measured with three AUDIT items that captured drink-related guilt, drink-related memory loss and alcohol-related injury. The alcohol-related injury item score was coded as a binary measure, with lifetime or past year involvement in an alcohol-related injury both coded as one, and reporting no history of alcohol-related injury coded as zero. Scores for these three variables were summed to give a score for harmful effects of drinking, ranging from 0 to 10.

Concern from others about drinking was measured by a single AUDIT item asking if anyone, professional or personal, had expressed concerns about drinking. Participants were coded positive for ‘concern from others about drinking’ if they reported ever having received expressions of concern from others.

### Outcome variables

**Fig. 1:**
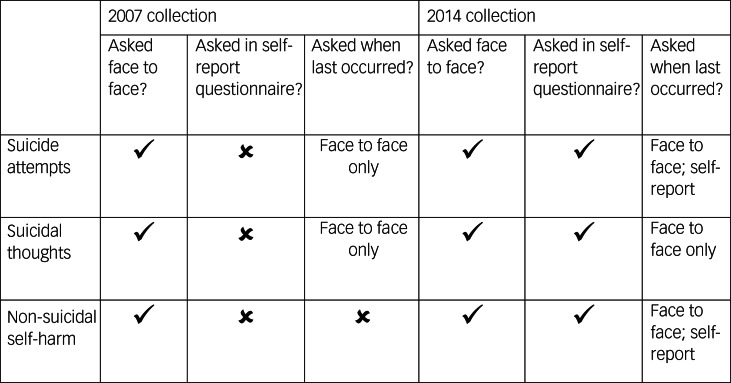
Description of how the outcome was asked across Adult Psychiatric Morbidity Survey collection points.

Suicide attempts, suicidal thoughts and non-suicidal self-harm were measured with standardised items as part of the Clinical Interview Schedule, Revised, in 2007 and 2014, and were separately asked in a self-completed questionnaire that utilised a computer-assisted self-completed interview in 2014. Participants were asked ‘Have you ever made an attempt to take your life, by taking an overdose of tablets or in some other way?’, ‘Have you ever thought of taking your life, even though you would not actually do it?’ and ‘Have you ever deliberately harmed yourself in any way but not with the intention of killing yourself?’. Those who answered positively to either of these questions were asked a follow-up question on when this had last occurred. Figure 1 outlines how these responses were collected across the APMS surveys. As there was no way of specifying past year non-suicidal self-harm in 2007, only the 2014 data were used for this outcome.

### Potential confounders

We selected ten sociodemographic and clinical covariates as potential confounders *a priori* based on associations with alcohol and with suicidal behaviour: gender,^27^ age,^27^ marital status,^27^ neighbourhood deprivation,^28^ educational qualification,^27^ employment status,^27^ any self-reported mental health problem since age 16 years,^19^ any past year drug use^29^ and number of chronic physical health conditions.^30^ Of these, the following were captured through interview questions: gender (self-identified male/female), age, marital status (married or cohabiting; single; widowed, separated or divorced), educational qualification (degree, A-levels or other non-degree qualification, up to GCSE or equivalent, no qualifications), employment status (employed, unemployed, not economically active) and history of any mental health problems since age 16 years (self-reported yes/no). Neighbourhood deprivation (Index of Multiple Deprivation score, grouped into quintiles) was allocated based on each participant's postal address. Any past year drug use was reported as part of a set of questions relating to drug use that was administered by computer-assisted self-completed interview. Number of chronic physical health conditions were summed based on the number of self-reported health problems reported from a list of 19 chronic health conditions. Further details on how we derived the variables capturing mental health problems, physical health problems and drug use are provided in Supplementary Table 2.

### Statistical analyses

We reported descriptive statistics, and presented associations between covariates and the primary exposures explored with chi-squared and *t*-tests, as appropriate. For main analyses, we conducted univariable analyses followed by multivariable analyses to adjust for the effects of ten specific potential confounders.

To investigate the association between alcohol use and our three outcomes, we conducted multivariable logistic regressions. For our main analysis, we used total AUDIT score (continuous) to investigate whether there is a linear association between AUDIT score and outcomes. To investigate associations of our three outcomes with other domains of alcohol use, we ran models with categorical measures of drinking risk category (low-/moderate-/high-risk alcohol use) and of five specific domains of alcohol use. Interaction tests were used to explore whether age and gender modified any associations between alcohol use and outcomes.

All analyses were performed with Stata for Windows version 16.1.^31^ All analyses were weighted using the weighting provided with the original APMS survey data to ensure that the sample was as representative of the general population as possible. Missing data were imputed by using multiple imputation by chained equation.^32^ To impute, we used all variables from the final model, and included ethnicity as an auxiliary variable; ten data-sets were imputed. We ran an interaction test for the outcomes with survey year as the moderating variable, and no difference was detected, thus indicating no detectable difference in this relationship between the two samples and supporting their amalgamation. In a sensitivity analysis, we re-ran our main models on the complete-case sample. We ran a further *post hoc* sensitivity analysis to explore the confounding effect of self-reported mental health conditions as distinct from that of all other listed confounding variables. To do this, we ran a model adjusted for all confounders apart from self-reported mental health conditions, and then added mental health conditions to the final fully adjusted model.

## Results

A total of 14 949 participants were included in our study. The majority of participants were married (62%), employed (59%) and of White ethnicity (89%). The mean age of the sample was 47.2 years (s.d. = 19.1). The median AUDIT score of the sample was 3 (IQR = 1–6). Prevalence of past year suicidal thoughts and past year suicide attempt was 4.6% and 0.8%, respectively. Further demographic and clinical characteristics are provided in Table 1. Associations between total AUDIT score by confounding factors and outcomes is presented in Supplementary Table 3. Of those who completed the APMS surveys, data were missing for 3%. A comparison of those with and without missing data is shown in Supplementary Table 4.

**Table 1:** Summary of sample sociodemographic and clinical characteristics^a^

Descriptive characteristic	Sample (*n* = 14 949)
Gender, *n* (%)	
Male	6255 (49)
Female	8694 (51)
Missing	−
Age, years, mean (s.d.)	47.2 (19.1)
Missing	−
Marital status, *n* (%)	
Married or cohabiting	8270 (62)
Single	3016 (23)
Widowed, divorced or separated	3663 (15)
Missing	−
Neighbourhood deprivation (IMD score), *n* (%)	
<8.35 (Least deprived)	2972 (19)
8.35–13.72	3189 (21)
13.72–21.16	3030 (20)
21.16–34.21	2844 (19)
>34.21 (Most deprived)	2914 (20)
Missing	−
Highest educational qualification, *n* (%)	
Degree	3174 (23)
A-levels or other non-degree qualification	3282 (24)
Up to GCSE or equivalent	4122 (29)
No qualifications	4121 (24)
Missing, *n*	250
Employment status, *n* (%)	
Employed	7985 (59)
Unemployed	382 (3)
Not economically active	6582 (38)
Missing	−
Ethnicity, *n* (%)	
White	13 620 (89)
Black	385 (3)
South Asian	556 (5)
Mixed or other	310 (3)
Missing, *n*	78
Any self-reported mental health problem since age 16 years, *n* (%)	5391 (33)
Missing, *n*	3
Any past year drug use, *n* (%)	995 (9)
Missing, *n*	222
Number of chronic health conditions, median (IQR)	1 (0.3)
Missing, *n*	6
AUDIT score, median (IQR)	3 (1.6)
Missing, *n*	19
Suicidal thoughts in the past year, *n* (%)	730 (4.6)
Missing, *n*	23
Suicide attempt in the past year, *n* (%)	117 (0.8)
Missing, *n*	20
Non-suicidal self-harm in the past year,^b^ *n* (%)	118 (1.8)
Missing, *n*	7

IMD, Index of Multiple Deprivation; IQR, interquartile range; AUDIT, Alcohol Use Disorders Identification Test.

a. Sample sizes and *n* are raw (unweighted); means, medians and frequencies are all weighted.

b. Based on a sample of 7539.

### Association between total AUDIT score and suicidal behaviour

Results for the univariable and multivariable analyses assessing the relationship between total AUDIT score and suicidal behaviour are shown in Table 2.

**Table 2:** Association between total Alcohol Use Disorders Identification Test scores and suicidal behaviours

	Unadjusted model, odds ratio (95% CI)	Fully adjusted model,^a^ odds ratio (95% CI)
Suicide attempts	1.11 (1.08–1.14)	1.06 (1.03–1.09)
Suicidal thoughts	1.07 (1.06–1.10)	1.05 (1.03–1.07)
Non-suicidal self-harm	1.08 (1.05–1.12)	1.04 (1.01–1.08)

*N* = 14 949.

a. Model adjusted for gender, age, marital status, neighbourhood deprivation, educational qualification, employment status, self-reported mental health problems, past year drug use and number of physical health conditions.

Using the total AUDIT score scale as a continuous measure, univariable logistic regression showed evidence of a linear relationship between AUDIT score and suicide attempt, with the odds of having made a suicide attempt in the past year increasing by 11% for every point increase in AUDIT score (odds ratio 1.11, 95% CI 1.08–1.14). This association was attenuated when sociodemographic and clinical covariates were added to the model, but strong evidence of an association remained (adjusted odds ratio 1.06, 95% CI 1.03–1.09). Similarly, there was evidence of a relationship between AUDIT score and suicidal thoughts in both the unadjusted (odds ratio 1.07, 95% CI 1.06–1.10) and fully adjusted model (adjusted odds ratio 1.05, 95% CI 1.03–1.07).

For the outcome non-suicidal self-harm, only data collected as part of the 2014 APMS (*n* = 7539) was used, as it was not possible to ascertain past year non-suicidal self-harm in the 2007 collection sample. Using the AUDIT score as a continuous measure, there was an association between total AUDIT score and non-suicidal self-harm in the unadjusted model (odds ratio 1.08, 95% CI 1.05–1.12), and this effect was weakened but remained after adjusting for potential confounders (odds ratio 1.04, 95% CI 1.01–1.08).

Neither gender nor age modified any of these associations (see Supplementary Tables 5 and 6, Supplementary Figs 1–3).

### Association between AUDIT score risk categories and suicidal behaviour

Results for the univariable and multivariable analyses assessing the relationship between grouped AUDIT score and suicidal behaviour are shown in Table 3. The odds of suicidal behaviour increased across alcohol use risk groups for all outcomes in the unadjusted models, with the highest risk group showing strong evidence of an association with suicidal behaviour outcomes. Adjusting for confounders attenuated the odd ratios for all outcomes. Following adjustment, evidence of an effect remained for suicidal thoughts and suicide attempts, but not for non-suicidal self-harm.

**Table 3:** Association between Alcohol Use Disorders Identification Test score risk categories and suicidal behaviours

		Unadjusted model, odds ratio (95% CI)	*P*-trend	Fully adjusted model, odds ratio (95% CI)	*P*-trend
Suicide attempts	Low-risk alcohol use	1 (Reference)	<0.001	1 (Reference)	0.005
	Moderate-risk alcohol use	1.80 (1.03–3.15)		1.46 (0.77–2.75)	
	High-risk alcohol use	9.25 (5.32–16.12)		3.47 (1.65–7.27)	
Suicidal thoughts	Low-risk alcohol use	1 (Reference)	<0.001	1 (Reference)	<0.001
	Moderate-risk alcohol use	1.41 (1.12–1.80)		1.25 (0.96–1.63)	
	High-risk alcohol use	6.01 (4.45–8.12)		3.96 (2.65–5.91)	
Non-suicidal self-harm	Low-risk alcohol use	1 (Reference)	<0.001	1 (Reference)	0.211
	Moderate-risk alcohol use	1.56 (0.92–2.64)		0.99 (0.55–1.80)	
	High-risk alcohol use	4.93 (2.45–9.94)		2.02 (0.88–4.62)	

*N* = 14 949. Model adjusted for gender, age, marital status, neighbourhood deprivation, educational qualification, employment status, self-reported mental health problems, past year drug use and number of physical health conditions. Alcohol Use Disorders Identification Test scores: 0–7, low-risk alcohol use; 8–15, moderate-risk alcohol use; ≥16, high-risk alcohol use.

### Domains of alcohol use and suicidal behaviours

Results from our analyses testing the association between different domains of alcohol use and suicidal behaviours are presented in Table 4. Daily or almost daily binge drinking, dependence symptom score and harmful effects of alcohol use score were all associated with increased odds of suicide attempts, suicidal thoughts and non-suicidal self-harm. The remaining alcohol use domains were inconsistently associated with suicidal behaviour. For drinking quantity and frequency, the highest consumption group had increased odds of suicide attempts and suicidal thoughts, but no associations with non-suicidal self-harm were observed. Similarly, concern from others about drinking was associated with suicide attempts and suicidal thoughts, but not with non-suicidal self-harm. Conversely, daily or almost daily binge drinking was associated with all three outcomes; weekly binge drinking was associated with increased odds of suicidal thoughts, but not with suicide attempts or non-suicidal self-harm; and monthly binge drinking was not associated with any outcomes.

**Table 4:** Association between alcohol use domains and suicidal behaviours

	Suicide attempts		Suicidal thoughts		Non-suicidal self-harm	
		Unadjusted model, odds ratio (95% CI)	Fully adjusted model, odds ratio (95% CI)	Unadjusted model, odds ratio (95% CI)	Fully adjusted model, odds ratio (95% CI)	Unadjusted model, odds ratio (95% CI)	Fully adjusted model, odds ratio (95% CI)
Drinking quantity and frequency	Light drinking	1 (Reference)	1 (Reference)	1 (Reference)	1 (Reference)	1 (Reference)	1 (Reference)
	Moderate drinking	0.96 (0.36–2.55)	2.07 (0.75–5.68)	0.76 (0.49–1.16)	1.08 (0.70–1.66)	0.10 (0.03–0.45)	0.23 (0.05–1.01)
	Hazardous drinking	0.58 (0.23–1.44)	0.83 (0.30–2.32)	0.83 (0.58–1.20)	0.97 (0.65–1.45)	0.75 (0.27–2.05)	1.11 (0.40–3.15)
	Harmful drinking	1.55 (0.64–3.73)	1.31 (0.46–3.70)	1.66 (1.18–2.33)	1.60 (1.10–2.32)	2.55 (1.17–5.53)	2.44 (0.92–6.47)
	Probable dependence	5.39 (2.74–10.58)	3.66 (1.55–8.66)	3.47 (2.32–5.21)	2.61 (1.59–4.29)	2.26 (0.92–5.54)	1.67 (0.68–4.10)
Binge drinking	Less than monthly or never	1 (Reference)	1 (Reference)	1 (Reference)	1 (Reference)	1 (Reference)	1 (Reference)
	Monthly	1.66 (0.87–3.16)	1.21 (0.64–2.29)	1.54 (1.17–2.03)	1.29 (0.95–1.76)	1.51 (0.75–3.05)	0.98 (0.43–2.25)
	Weekly	1.84 (0.97–3.47)	1.48 (0.71–3.09)	1.61 (1.22–2.14)	1.44 (1.04–1.98)	1.94 (0.96–3.90)	1.29 (0.61–2.70)
	Daily or almost daily	10.10 (4.93–20.66)	5.59 (2.37–13.20)	4.78 (3.08–7.41)	2.83 (1.72–4.64)	5.22 (2.17–12.61)	4.69 (1.54–14.32)
Dependence symptoms		1.43 (1.33–1.53)	1.20 (1.10–1.30)	1.36 (1.29–1.44)	1.19 (1.12–1.26)	1.32 (1.20–1.45)	1.13 (1.00–1.29)
Harmful effects of drinking		1.56 (1.43–1.70)	1.27 (1.13–1.42)	1.44 (1.36–1.53)	1.24 (1.16–1.34)	1.42 (1.27–1.59)	1.20 (1.01–1.43)
Others concerned about drinking		5.91 (3.32–10.53)	2.99 (1.45–6.16)	4.06 (2.99–5.51)	2.52 (1.75–3.64)	2.64 (1.28–5.45)	1.55 (0.74–3.28)

Model adjusted for gender, age, marital status, neighbourhood deprivation, educational qualification, employment status, self-reported mental health problems, past year drug use and number of physical health conditions. Drinking quantity and frequency: light drinking (0–7), moderate drinking (7–13), hazardous drinking (14–20), harmful drinking (21–30), probable dependence (≥30).

### Sensitivity analyses

Complete data was available for 14 458 participants. There were no substantial differences between the main findings, which used imputation to account for missing data, and the finding from sensitivity analysis using complete-case analysis (Supplementary Tables 7–9). In models exploring the contribution of self-reported mental health conditions to confounding of the main association, as distinct from that of other confounders, it was apparent that prior mental health accounted for only a small proportion of attenuation relative to other confounding factors (results not shown).

## Discussion

### Main findings

In this large population-based survey, we found evidence to support the existence of a linear association between harmful alcohol use, as measured by the total AUDIT score, and suicide attempts, suicidal thoughts and non-suicidal self-harm. We were also able to investigate whether risk categories and specific domains of alcohol use were differently associated with suicidal behaviour. These latter analyses revealed that, as anticipated, markers of extreme and harmful use of alcohol (dependence symptoms, harmful effects of alcohol, concern from others about drinking and daily or almost daily binge drinking) were all associated with suicidal behaviours. No other consistent patterns emerged between the outcomes and the other recorded domains of alcohol use. Upon adjustment for a range of *a priori* demographic, social and psychiatric confounders, we observed a noticeable attenuation of the strength of the association between alcohol use and suicidal behaviour, although independent effects remained even in the mutually adjusted model. This suggests that there are possible direct associations between harmful alcohol use and self-harm or suicidal behaviour, and between specific alcohol-related behaviours and these outcomes, which are not explained by these other observed factors.

### Interpretation of findings

Previous research on linear associations between harmful alcohol use and suicidal behaviour has reported inconsistent findings, perhaps because of the heterogeneity of measures, drinking cultures and study populations. Most studies in this area report on suicidal thoughts and death by suicide as outcomes, with fewer reporting on the relationship between alcohol use and suicide attempt or non-suicidal self-harm. To our knowledge, only one previous study has looked at the linear relationship between alcohol use and suicidal behaviour in a general population adult sample.^12^ This cross-sectional study in a Korean adult sample reported no clear relationship between AUDIT scores (categorised by increasing risk groups) and suicidal thoughts and suicide attempts in men, but evidence of a relationship in women.^12^ This is not wholly consistent with our findings, which did not reveal any clear gender interactions. Gender differences in the relationship between alcohol use and suicide have been reported,^20^ although this finding is not consistent, even at the level of alcohol dependence.^33^ In a UK-based psychiatric in-patient sample, higher AUDIT scores were associated with suicidal behaviour,^7^ as consistent with our findings, although this study compared differing cut-off points in a binary measure rather than our more nuanced use of different domains of the AUDIT tool.

We found no statistical evidence for an association between drinking quantity and frequency and suicide attempts, although when investigated categorically, the highest consumption group did show evidence of an increased odds of suicidal behaviour. This finding appears to be consistent with the few studies that have attempted to investigate quantity and frequency of alcohol consumption and risk of suicidal behaviour. A Korean study investigating the relationship between frequency of drinking and quantity consumed per drinking day and risk of suicidal behaviour found no association.^12^ A USA-based study that used a representative general population sample found no association between alcohol use frequency (measured as total number of drinking days in the past year) and suicidal ideation or attempt.^34^ Similarly, another USA-based study found no association between average drinks consumed and suicidal death among a sample of professional men aged 40–75 years.^18^ In a sample of elderly Australian adults, consuming alcohol less frequently, but in greater quantities (i.e. bingeing), increased risk of suicidal attempt.^35^ Binge drinking has been shown to be associated with suicidal behaviour.^36^ In our English sample, it appeared that weekly binge drinking did not increase the odds of either suicide attempt or suicidal ideation. This could be a reflection of the culture of heavy drinking in the UK, with one of the highest frequencies of binge drinking in Europe.^37^ Only one previous study has investigated the specific domains of alcohol use used in our analysis. In a Korean sample, increased frequency of drinking blackout was associated with suicidal ideation and attempt in men,^12^ which corresponds with the increased odds of suicidal thoughts associated with harmful effects of drinking score seen in our sample. These domains of dependence behaviours and harmful effects of drinking alcohol are those that may be more meaningful to the general public, and could be identified as clinical targets when working with patients to reduce risky drinking behaviour. Our analyses suggest that dependence-related behaviours and harmful effects of alcohol use, rather than consumption patterns, may potentially be the key underlying factor in the relationship between alcohol use and suicidal behaviour.

### Strengths and limitations

The strengths of our study include the use of a nationally representative sample, the use of a validated scale capturing alcohol use, our novel approach to exploring the associations of different domains of the AUDIT questionnaire with suicidal behaviour, and the use of multiple imputation to address missing data.

The main limitation of this study is the cross-sectional nature of APMS survey data, which means we cannot be certain about the temporality of events nor infer causation. We have been careful to limit the timeframe for outcomes by only considering past year suicidal thoughts, suicide attempts and non-suicidal self-harm, which relate closely to the timeframe for the AUDIT questionnaire of the past year. However, a longitudinal analysis is needed to rule out any potential reverse causation for the associations observed. Although it is possible that samples from 2007 and 2014 might overlap, this is likely to be negligible, given random sampling for both. We acknowledge the possibility of residual confounding as we lacked variables that allowed us to identify and control for previous suicide attempt, one of the most recognised predictors of suicide attempt, which is also associated with harmful alcohol use. We recognise that mental health conditions could be considered as a confounder or a mediator in the association between alcohol use and suicidal behaviours. Because of the cross-sectional nature of this data-set, it was not possible to conduct a mediation analysis, and future research using an appropriate longitudinal data-set should investigate this in a population-based sample. We also acknowledge the potential for underascertainment of outcomes, as suicidal thoughts and attempt were both reported in the CIS-R face-to-face interviews. It is possible that some people, and particularly those from backgrounds with more stigmatising views toward suicidal behaviours, would choose not to disclose such sensitive information. Lastly, our use of specific domains of alcohol use as exposures deviates from the established subscales of the AUDIT questionnaire. Recent reviews of the composition of the AUDIT questionnaire challenge the two-factor structure,^38,39^ the conclusions of which support our breakdown of the AUDIT items into separate domains. We see this pragmatic breakdown of the AUDIT questionnaire as a strength of this study, in that it creates a series of clinically useful components of alcohol use, which would be meaningful to members of the public. However, we acknowledge that using this set of outcomes and conducting multiple testing increased the likelihood of reporting a type 1 error.

### Implications for policy, practice and future research

Alcohol misuse is a compelling modifiable risk factor for both suicide and non-suicidal self-harm, and effective treatment for alcohol misuse and dependence exists.^39,40,41^ Our study provides further support that populations who are at increasing risk of alcohol misuse are also at a higher risk of suicidal behaviour. Although this study did not measure suicide as an outcome, attempted suicide and non-suicidal self-harm are established risk factors for future suicide attempts and suicidal death.^8,42,43^ Regular monitoring of alcohol use by clinicians, and inquiring about suicidal behaviour in anyone who reports harmful drinking or concerning changes in their drinking, could be an effective measure for identifying people at high risk of suicide, requiring selective suicide prevention. Furthermore, our analyses identified simple domains of alcohol misuse, such as others’ concerns about drinking, which can be readily understood by the public and targeted, perhaps through motivational interviewing,^40^ to reduce risk of future suicidal behaviour.

Longitudinal research is needed to further support these findings empirically and ascertain potential causal associations, in addition to gaining insights into which groups of alcohol users in the general population would be most at risk for suicidal behaviours. Shifting the research focus from binge drinking to other dimensions of alcohol use may be warranted, subject to the availability of sufficiently nuanced data. Qualitative research exploring drinking motives and contexts for alcohol consumption may further enhance our understanding of the role of alcohol use behaviours and links with suicidal and self-harming behaviour.

## Data Availability

The data that support the findings of this study are held by NatCen Social Research and NHS Digital, and access is available on request through the UK Data Request Service.

## Supplementary material

For supplementary material accompanying this paper visit http://doi.org/10.1192/bjo.2022.594. click here to view supplementary material
