# Supplementary material for: Alcohol use and its association with suicide attempt, suicidal thoughts and non-suicidal self-harm in two successive, nationally representative English household samples
Source: BJPsych Open. 2022 Nov 3;8(6):e192. doi: 10.1192/bjo.2022.594 (PMC9634588; doi:10.1192/bjo.2022.594)
Supplement: Supplementary file 1 [file S2056472422005944sup001.docx]

Table S1: AUDIT items and response options in Adult Psychiatric Morbidity Survey (APMS) dataset

| **AUDIT item** | **Response options** |
| --- | --- |
| In the last 12 months, how often have you had a drink containing alcohol? | Never; Monthly; Two to four times a month; Two to three times a week; Four or more times a week |
| How many standard drinks containing alcohol do you have on a typical day when you are drinking? | One or two; Three or four; Five or six; Seven, eight or nine; Ten or more |
| How often do you have six or more drinks on one occasion? | Never; Less than monthly; Monthly; Weekly; Daily or almost daily |
| How often during the last year have you found that you were not able to stop drinking once you had started? | Never; Less than monthly; Monthly; Weekly; Daily or almost daily |
| How often during the last year have you failed to do what was normally expected from you because of drinking? | Never; Less than monthly; Monthly; Weekly; Daily or almost daily |
| How often during the last year have you needed a drink first thing in the morning to get yourself going after a heavy drinking session? | Never; Less than monthly; Monthly; Weekly; Daily or almost daily |
| How often during the last year have you had a feeling of guilt or remorse after drinking? | Never; Less than monthly; Monthly; Weekly; Daily or almost daily |
| How often during the last year have you been unable to remember what happened the night before because you had been drinking? | Never; Less than monthly; Monthly; Weekly; Daily or almost daily |
| Have you or someone else been injured as a result of your drinking? | Yes, but not in the last year; Yes, during the last year; No |
| Has a relative, a friend, or a doctor or other health worker been concerned about your drinking or suggest you cut down? | Yes, but not in the last year; Yes, during the last year; No |

Table S2: Further description of mental health, physical health and drug use variables in Adult Psychiatric Morbidity Survey (APMS) dataset

| Variable | Description |
| --- | --- |
| Any self-reported mental health problems since age 16 | Includes self-report of the following 15 mental health conditions: depression; anxiety; post-traumatic stress disorder; phobia; panic attacks; attention deficit hyperactivity disorder; bipolar disorder; post-natal depression; seasonal affective disorder; eating disorder; personality disorder; ‘nervous breakdown’; psychosis; obsessive-compulsive disorder; any other anxiety disorder; and any other neurotic/emotional disorder (any explicit reports of alcohol use disorder diagnosis were not included in this measure) |
| Past year drug use | Includes use of cannabis; amphetamines; cocaine; crack; ecstasy; heroin; acid/LSD; magic mushrooms; methadone/physeptone; tranquilisers; amyl nitrate poppers; anabolic steroids; glue/solvents/aerosols |
| Chronic health conditions | Includes cancer; diabetes; epilepsy/fits; migraine/frequent headaches; dementia/Alzheimer’s disease; stroke; heart attack/angina; high blood pressure; arthritis; bone, back, joint or muscle problems; skin problems; cataracts/eyesight problems; ear/hearing problems; bronchitis/emphysema; bowel/colon problems; other heart/blood vessel/circulatory system; other bone/joint/muscle problems; renal problems; other nervous system disorders. |

Table S3: Associations between key variables and total AUDIT score

| ***Descriptive characteristic*** | ***Sample***  *N (%)** | ***Total AUDIT score***  *Median (IQR)** | ***P value*** |
| --- | --- | --- | --- |
| **Total** | 14,461 | 3 (1, 7) |  |
| **Potential confounders** |  |  |  |
| **Gender** |  |  | <0.001 |
| Male | 6,002 (49) | 4 (2,8) |  |
| Female | 8,459 (51) | 3 (0,5) |  |
| **Age *mean (sd)*** | 47.13 (19.1) | -0.05^b^ | <0.001 |
| **Marital Status** |  |  | <0.001 |
| Married or cohabiting | 8,046 (62) | 3 (1,6) |  |
| Single | 2,912 (23) | 4 (1,9) |  |
| Widowed, divorced or separated | 3,503 (14) | 2 (0,5) |  |
| **Neighbourhood deprivation (IMD Score)** |  |  | 0.134 |
| <8.35 (Least deprived) | 2,883 (20) | 4 (2,6) |  |
| 8.35-13.72 | 3,103 (21) | 4 (1,7) |  |
| 13.72-21.16 | 2,933 (20) | 4 (1,7) |  |
| 21.16-34.21 | 2,743 (19) | 3 (1,7) |  |
| >34.21 (Most deprived) | 2,799 (20) | 3 (0,6) |  |
| **Highest educational qualification** |  |  | <0.001 |
| Degree | 3,135 (23) | 4 (2,7) |  |
| A-levels or other non-degree qualification | 3,243 (24) | 4 (1,7) |  |
| Up to GCSE or equivalent | 4,058 (29) | 4 (1,7) |  |
| No qualifications | 4,025 (23) | 2 (0,5) |  |
| **Employment status** |  |  | <0.001 |
| Employed | 7,806 (60) | 2 (4,7) |  |
| Unemployed | 366 (3) | 4 (0,8) |  |
| Not economically active | 6,274 (37) | 2 (0,5) |  |
|  |  |  |  |
| **Any self-reported mental health problem since age 16** |  |  | 0.005 |
| Yes | 5,541 (38) | 3 (1,6) |  |
| No | 8,920 (62) | 3 (1,6) |  |
| **Any past year drug use *n (%)*** |  |  | <0.001 |
| Yes | 983 (9) | 8 (4,12) |  |
| No | 13,478 (91) | 3 (1,6) |  |
| **Number of chronic health conditions *median (IQR)*** | 1 (0,3) | -0.24^b^ | <0.001 |
| **Outcomes** |  |  |  |
| **Suicidal thoughts in the past year** |  |  | <0.001 |
| Yes | 689 (4.5) | 4 (1,10) |  |
| No | 13,772 (95.5) | 3 (1,6) |  |
| **Suicide attempt in the past year** |  |  | <0.001 |
| Yes | 117 (0.8) | 6 (0,14) |  |
| No | 14,344 (99.2) | 3 (1,6) |  |
| **Self harm in the past year^a^ *[based on sample of 7275]*** |  |  | 0.001 |
| Yes | 111 (1.8) | 5 (0,10) |  |
| No | 7,164 (98.2) | 3 (0,6) |  |

^a^Based on a sample of 7275 ^b^Co-efficient from linear regression model *Unless otherwise specified

Table S4: Demographic and clinical characteristics of included and excluded participants

| ***Descriptive characteristic*** | ***Missing data (n=488, 3%)*** | ***Complete case***  ***(n=14,461, 97%)*** | ***p-value*** |
| --- | --- | --- | --- |
| **Potential confounders** |  |  |  |
| **Gender *n (%)*** |  |  | *0.001* |
| Male | 255 (57) | 6,002 (49) |  |
| Female | 236 (43) | 8,459 (51) |  |
| **Age *mean (sd)*** | 49.32 (20.42) | 47.13 (19.05) | *0.046* |
| **Marital Status *n (%)*** |  |  | *0.006* |
| Married or cohabiting | 224 (56) | 8,046 (62) |  |
| Single | 104 (24) | 2,912 (23) |  |
| Widowed, divorced or separated | 160 (20) | 3,503 (14) |  |
| **Neighbourhood deprivation (IMD Score) *n (%)*** |  |  | *0.037* |
| <8.35 (Least deprived) | 89 (17) | 2,883 (20) |  |
| 8.35-13.72 | 86 (17) | 3,103 (21) |  |
| 13.72-21.16 | 97 (19) | 2,933 (20) |  |
| 21.16-34.21 | 101 (20) | 2,743 (19) |  |
| >34.21 (Most deprived) | 115 (26) | 2,799 (20) |  |
| **Highest educational qualification *n (%)*** |  |  | *<0.001* |
| Degree | 39 (20) | 3,135 (23) |  |
| A-levels or other non-degree qualification | 39 (15) | 3,243 (24) |  |
| Up to GCSE or equivalent | 64 (29) | 4,058 (29) |  |
| No qualifications | 96 (36) | 4,025 (23) |  |
| *Missing* | 250 | - |  |
| **Employment status *n (%)*** |  |  | *<0.001* |
| Employed | 172 (41) | 7,806 (60) |  |
| Unemployed | 16 (6) | 366 (3) |  |
| Not economically active | 300 (54) | 6,274 (37) |  |
| **Ethnicity *n (%)*** |  |  | *0.642* |
| White | 393 (90) | 13,227 (89) |  |
| Black | 12 (4) | 373 (3) |  |
| South Asian | 10 (4) | 546 (5) |  |
| Mixed or other | 7 (2) | 303 (3) |  |
| *Missing* | 66 | - |  |
| **Any self-reported mental health problem since age 16 *n(%)*** | 234 (49) | 5,541 (38) | *<0.001* |
| **Any past year drug use *n (%)*** | 12 (5) | 983 (9) | *0.114* |
| *Missing* | 222 | - |  |
| **Number of chronic health conditions *median (IQR)*** | 2 (1,3) | 1 (0,3) | *0.010* |
|  |  |  |  |
| **AUDIT score *median (IQR)*** | 4 (1,6) | 3 (1, 7) | *0.117* |
| **Outcome measures** |  |  |  |
| **Suicidal thoughts in the past year *n (%)*** | 41 (9.5) | 689 (4.5) | *<0.001* |
| *Missing* | 23 | - |  |
| **Suicide attempt in the past year *n (%)*** | 12 (3.0) | 117 (0.8) | *<0.001* |
| *Missing* | 20 | - |  |
| **Self harm in the past year^b^ *n (%) [based on sample of 7275]*** | 7 (1.9) | 111 (1.2) | *0.826* |

Table S5: Association between total AUDIT score and suicidal behaviours by gender: stratum-specific analyses

|  | **Male** | **Female** | **Interaction term**  **p-value** |
| --- | --- | --- | --- |
|  | **Unadjusted OR (95% CI)** | **Unadjusted OR (95% CI)** |  |
| **Suicide attempts** | 1.11 (1.06-1.16) | 1.14 (1.09-1.18) | *0.533* |
| **Suicidal thoughts** | 1.08 (1.06-1.11) | 1.10 (1.07-1.12) | *0.361* |
| **Non-suicidal self-harm** | 1.07 (1.02-1.13) | 1.12 (1.07-1.17) | *0.229* |

Table S6: Interaction of age with AUDIT score and suicidal behaviour

|  | **Interaction term**  **p-value** |
| --- | --- |
|  |  |
| **Suicide attempts** | *0.282* |
| **Suicidal thoughts** | *0.339* |
| **Non-suicidal self-harm** | *0.490* |


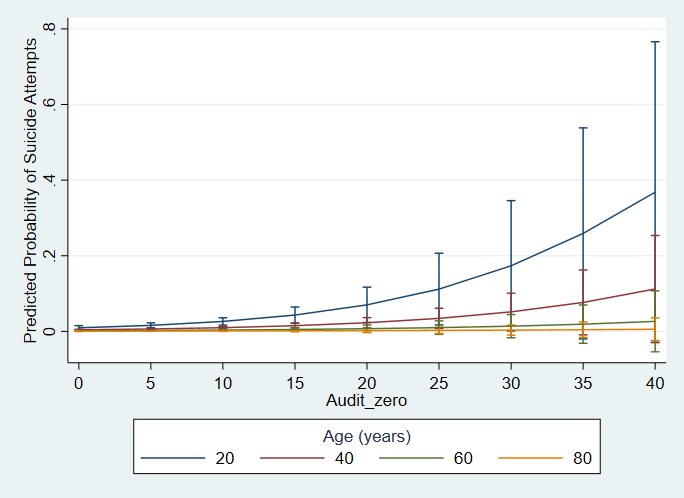


Fig S1: Margins plot demonstrating interaction of age with AUDIT score and suicide attempts at selected age points


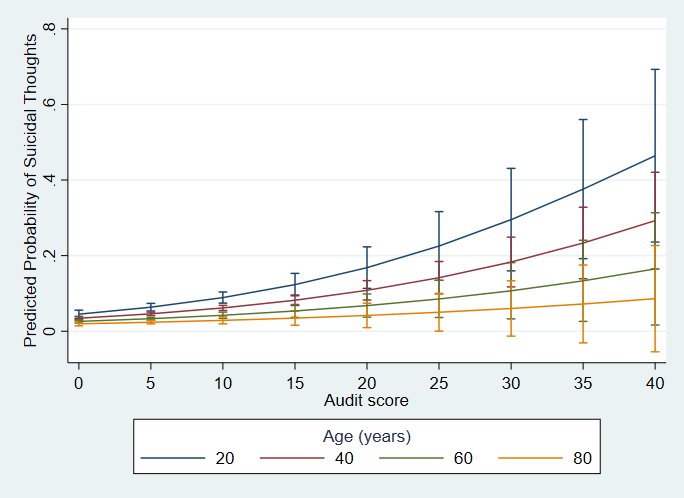


Fig S2: Margins plot demonstrating interaction of age with AUDIT score and suicidal thoughts at selected age points


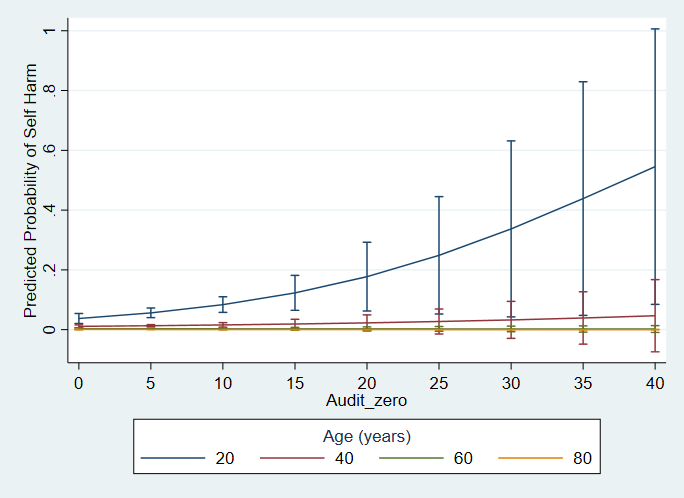


Fig S3: Margins plot demonstrating interaction of age with AUDIT score and self-harm at selected age points

Table S7: Sensitivity analysis: Association between total AUDIT scores and suicidal behaviours in complete cases

|  | **Unadjusted model**  **OR (95% CI)** | **Fully adjusted model**  **OR (95% CI)** |
| --- | --- | --- |
| **Suicide attempts** | 1.11 (1.08-1.15) | 1.06 (1.03-1.10) |
| **Suicidal thoughts** | 1.08 (1.06-1.09) | 1.04 (1.03-1.06) |
| **Non-suicidal self-harm** | 1.08 (1.05-1.12) | 1.04 (1.00-1.09) |

N=14,461. Model adjusted for gender, age, marital status, neighbourhood deprivation, educational qualification, employment status, self-reported mental health problems, past year drug use, and number of physical health conditions.

Table S8: Sensitivity analysis: Association between AUDIT score risk categories and suicidal behaviour in complete cases

|  |  | **Unadjusted model**  **OR (95% CI)** | **P trend** | **Fully adjusted model**  **OR (95% CI)** | **P trend** |
| --- | --- | --- | --- | --- | --- |
| **Suicide attempts** | **Low risk drinking** | *1 (ref)* | <0.001 | *1 (ref)* | 0.001 |
|  | **Moderate risk drinking** | 1.69 (0.93-3.08) |  | 1.37 (0.71-2.67) |  |
|  | **High risk drinking** | 9.37 (5.21-16.88) |  | 3.70 (1.68-8.16) |  |
| **Suicidal thoughts** | **Low risk drinking** | *1 (ref)* | <0.001 | *1 (ref)* | <0.001 |
|  | **Moderate risk drinking** | 1.38 (1.08-1.77) |  | 1.20 (0.92-1.57) |  |
|  | **High risk drinking** | 5.63 (4.10-7.73) |  | 3.82 (2.49-5.87) |  |
| **Non-suicidal self-harm** | **Low risk drinking** | *1 (ref)* | <0.001 | *1 (ref)* | 0.10 |
|  | **Moderate risk drinking** | 1.45 (0.84-2.51) |  | 0.88 (0.47-1.63) |  |
|  | **High risk drinking** | 4.82 (2.29-10.13) |  | 2.08 (0.85-5.06) |  |

Model adjusted for gender, age, marital status, neighbourhood deprivation, educational qualification, employment status, self-reported mental health problems, past year drug use, and number of physical health conditions AUDIT 0-7: Low risk alcohol use; AUDIT 8-15: Moderate risk alcohol use; AUDIT 16+: High risk alcohol use

Table S9: Sensitivity analyses: Association between alcohol use domains and suicidal behaviours in complete cases

|  |  | **Suicide attempts** | | **Suicidal thoughts** | | **Non-suicidal self-harm** | |
| --- | --- | --- | --- | --- | --- | --- | --- |
|  |  | **Unadjusted model**  **OR (95% CI)** | **Fully adjusted model**  **OR (95% CI)** | **Unadjusted model**  **OR (95% CI)** | **Fully adjusted model**  **OR (95% CI)** | **Unadjusted model**  **OR (95% CI)** | **Fully adjusted model**  **OR (95% CI)** |
| **Drinking quantity and frequency^a^** | **Light drinking** | *1 (ref)* | *1 (ref)* | *1 (ref)* | *1 (ref)* | *1 (ref)* | *1 (ref)* |
|  | **Moderate drinking** | 1.01 (0.36-2.85) | 2.26 (0.78-6.49) | 0.79 (0.51-1.22) | 1.13 (0.73-1.75) | 0.05 (0.07-0.37) | 0.11 (.01-0.84) |
|  | **Hazardous drinking** | 0.65 (0.26-1.62) | 0.99 (0.36-2.74) | 0.82 (0.57-1.18) | 0.95 (0.64-1.42) | 0.63 (0.20-1.94) | 0.95 (0.30-2.96) |
|  | **Harmful drinking** | 1.74 (0.72-4.21) | 1.53 (0.53-4.40) | 1.76 (1.25-2.49) | 1.72 (1.18-2.52) | 2.41 (1.06-5.47) | 2.3 (0.80-6.57) |
|  | **Probable dependence** | 4.38 (2.07-9.26) | 3.14 (1.17-8.38) | 3.15 (2.04-4.88) | 2.46 (1.45-4.16) | 1.73 (0.59-5.03) | 1.40 (0.51-3.81) |
| **Binge drinking^b^** | **Less than monthly or never** | *1 (ref)* | *1 (ref)* | *1 (ref)* | *1 (ref)* | *1 (ref)* | *1 (ref)* |
|  | **Monthly** | 1.56 (0.79-3.08) | 1.14 (0.59-2.20) | 1.51 (1.14-2.00) | 1.24 (0.91-1.71) | 1.58 (0.78-3.20) | 1.01 (0.43-2.34) |
|  | **Weekly** | 2.06 (1.07-3.95) | 1.65 (0.77-3.54) | 1.61 (1.21-2.14) | 1.42 (1.03-1.96) | 1.97 (0.96-4.06) | 1.27 (0.59-2.75) |
|  | **Daily or almost daily** | 8.76 (4.11-18.65) | 5.43 (2.24-13.16) | 4.44 (2.81-7.02) | 2.75 (1.68-4.51) | 4.74 (1.75-12.83) | 5.77 (1.84-18.11) |
| **Dependence symptoms^c^** |  | 1.45 (1.34-1.56) | 1.24 (1.14-1.35) | 1.36 (1.28-1.44) | 1.19 (1.13-1.27) | 1.33 (1.20-1.48) | 1.16 (1.01-1.33) |
| **Harmful effects of drinking^c^** |  | 1.57 (1.43-1.71) | 1.28 (1.13-1.44) | 1.43 (1.35-1.52) | 1.23 (1.15-1.33) | 1.41 (1.25-1.60) | 1.20 (0.98-1.45) |
| **Others concerned about drinking^b^** |  | 5.67 (3.03-10.60) | 2.97 (1.37-6.47) | 3.69 (2.67-5.10) | 2.31 (1.57-3.40) | 2.31 (1.00-5.28) | 1.43 (0.62-3.28) |

Model adjusted for gender, age, marital status, neighbourhood deprivation, educational attainment, employment status, mental health problems, past year drug use, and number of physical health conditions. Drinking quantity and frequency: Light drinking (0-7), Moderate drinking (7-13), Hazardous drinking (14-20), Harmful drinking (21-30), Probable dependence (30+)

^a^n=14,419; non-suicidal self-harm n=7,254; ^b^n=14,456; non-suicidal self-harm n=7,274; ^c^n=14,458; non-suicidal self-harm n=7,273 ; ^d^n=14,459.
